# The Hemiptera (Insecta) of Canada: Constructing a Reference Library of DNA Barcodes

**DOI:** 10.1371/journal.pone.0125635

**Published:** 2015-04-29

**Authors:** Rodger A. Gwiazdowski, Robert G. Foottit, H. Eric L. Maw, Paul D. N. Hebert

**Affiliations:** 1 Biodiversity Institute of Ontario, University of Guelph, Guelph, Ontario, N1G 2W1, Canada; 2 Agriculture and Agri-Food Canada, Invertebrate Biodiversity—National Environmental Health Program, and Canadian National Collection of Insects, Arachnids and Nematodes, Ottawa, Ontario, K1A 0C6, Canada; University of Veterinary Medicine Hanover, GERMANY

## Abstract

DNA barcode reference libraries linked to voucher specimens create new opportunities for high-throughput identification and taxonomic re-evaluations. This study provides a DNA barcode library for about 45% of the recognized species of Canadian Hemiptera, and the publically available R workflow used for its generation. The current library is based on the analysis of 20,851 specimens including 1849 species belonging to 628 genera and 64 families. These individuals were assigned to 1867 Barcode Index Numbers (BINs), sequence clusters that often coincide with species recognized through prior taxonomy. Museum collections were a key source for identified specimens, but we also employed high-throughput collection methods that generated large numbers of unidentified specimens. Many of these specimens represented novel BINs that were subsequently identified by taxonomists, adding barcode coverage for additional species. Our analyses based on both approaches includes 94 species not listed in the most recent Canadian checklist, representing a potential 3% increase in the fauna. We discuss the development of our workflow in the context of prior DNA barcode library construction projects, emphasizing the importance of delineating a set of reference specimens to aid investigations in cases of nomenclatural and DNA barcode discordance. The identification for each specimen in the reference set can be annotated on the Barcode of Life Data System (BOLD), allowing experts to highlight questionable identifications; annotations can be added by any registered user of BOLD, and instructions for this are provided.

## Introduction

In this study, we present a DNA barcode library as a set of publicly available COI-5’ sequences linked to voucher specimens on the Barcode of Life Data System (BOLD) [1] that meet DNA barcode data standards [1], are identified to species listed in a taxonomic catalogue, and specified using a Digital Object Identifier (DOI, http://www.doi.org/). DNA barcode libraries make it possible sequences with their source specimens, which have been collected across time, habitats, identifiers, and institutions. Queries that use a reference set to make identifications rely on the quality of the reference material [2]. We propose that the definition of a set of DNA-barcoded specimens as a library, improves it as a basis for identifications in two main ways: #1) the specimens are explicitly defined for community review [3], allowing the library to be collaboratively improved [4]—this may often be the first time many specimens, identified to species, have been explicitly compared with each other [5]; and #2) query results can be rapidly compared with the recognized diversity of a taxon or region, based on prior taxonomic work.

### DNA barcode libraries as taxonomic tools

Because of its role as a repository for DNA barcode sequences and associated specimen data, BOLD, the Barcode of Life Data System [1] can serve as a workbench for constructing a reference library. Its effectiveness in supporting identifications [6,7] has been extended by introduction of the Barcode Index Number system (BIN) [8]. The BIN system employs a defined set of algorithms to group DNA barcode sequences into Operational Taxonomic Units (OTU)[9], which often correspond to species [8], and assigns each OTU with a unique identifier (a BIN number, linked to a DOI) [8]. BOLD [1] automatically generates a web page for each BIN that provides summary statistics on its member specimens, and enables the comparison and download of specimen records.

Aggregating specimens into presumptive species-level ‘bins’ has been practiced by entomologists for over a century. For example, C. V. Riley and H. H. Knight, leading hemipterists in the 19^th^ and early 20^th^ centuries, were among the first to advocate the storage of specimens in unit trays [10,11], a practice now the standard for separating insect specimens assigned to different species. Most unit trays in any collection contain specimens whose taxonomic provenance varies from certain (holotype) to ambiguous—all placed in a particular unit tray by curators or visiting specialists over time; the voucher specimens assembled on each BIN page are similarly accessed as a group. However, the use of a standardized algorithmic approach allows the merger of both identified and unidentified specimens in a way that makes it possible to visually compare DNA barcode diversity with nomenclatural diversity. The BIN page provides a clear report on the level of congruence (or discordance) among the DNA barcode records comprising a BIN and nomenclature [8] exposing taxonomic conflicts with the goal of aiding their resolution [6].

### Assembling DNA barcode libraries

The development of a DNA barcode library, for any group of organisms, requires the integration of taxonomic expertise, technologies, and cooperation among institutions [12,13], and helps to create new collaborative opportunities for constructing libraries at national and regional scales [14,15,16]. Prior efforts to develop DNA barcode libraries have adopted diverse approaches (S1 Table). Here, we build on these methods to describe the workflow employed in the construction of a DNA barcode library for Canadian species of Hemiptera [17]. As the fifth largest order of insects (after Coleoptera, Hymenoptera, Lepidoptera, Diptera), the Canadian fauna includes at least 3900 species of Hemiptera [16]. These taxa occur in both aquatic and terrestrial environments across Canada, and include some of its most damaging pest species as well as species used for biocontrol. In this manuscript, we provide an overview of the construction of the library, indicate methods for its use, and report on taxon coverage, taxon diversity, and DNA barcode-based genetic diversity within this group.

## Materials and Methods

### Data Release

We analyzed records for 54,280 specimens, which included 2671 species of Hemiptera from Canada whose specimen and DNA barcode information are available on BOLD. All records analyzed in this study are included in two projects on BOLD: ‘*Hemiptera of Canada—Main dataset parts I and II’* that can be accessed via two DOIs: 1) dx.doi.org/10.5883/DS-HECAMAIN; 2) dx.doi.org/10.5883/DS-HECAMN1. A subset of the above dataset, that is only new records for public release, are included in the projects: ‘*Hemiptera of Canada—New records for release parts I and II’* that can be accessed via two DOIs: 1) dx.doi.org/10.5883/DS-HECANEW; 2) dx.doi.org/10.5883/DS-HECANEW1. We consider a subset of records from the complete dataset (described in **Data Analyses**, below) as the current draft DNA barcode library for the Hemiptera of Canada. This subset includes 20,851 records that provide coverage for 1849 species assigned to 1867 BINs, which are available on BOLD in the project ‘*Hemiptera of Canada—DNA barcode library*’ via DOI dx.doi.org/10.5883/DS-HECALIB. All library specimen records, and their sequences are consistent with the DNA Barcode data standard [1] in terms of both sequence length (>500bp) and quality (less than 1% ns) as well as required specimen metadata. Many of the specimens in this data release are consistent, rather than strictly compliant, with the barcode data standard because they are based on high quality unidirectional reads (see **Specimen Collection and Processing)** rather than the conventional requirement for bidirectional reads.

**Fig 1:**
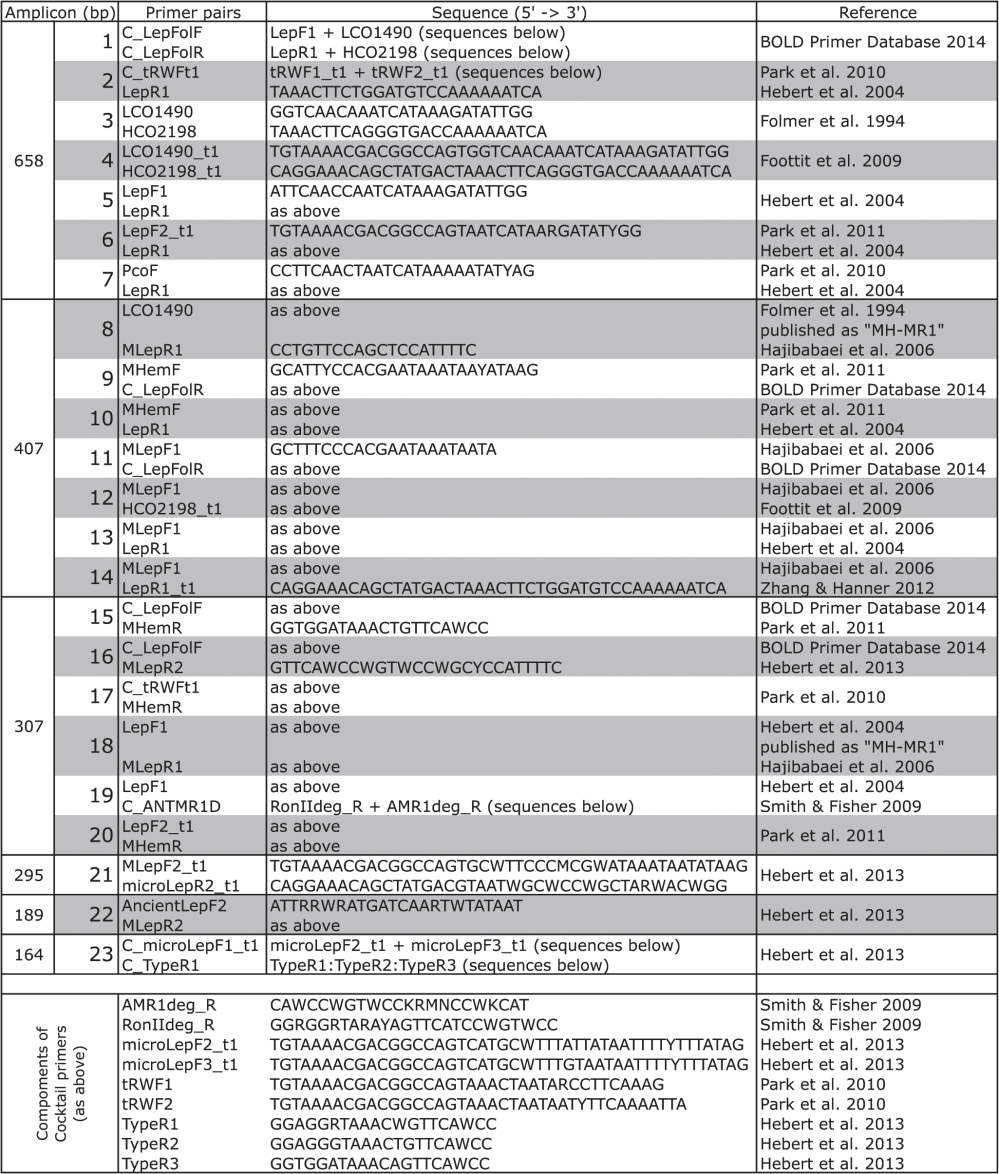
Primers amplifying the COI barcode region of Hemiptera examined in this study. This table pairs with the primer-use heatmap in Fig 2, where the sequentially shaded numbers in the left columns connect primer sequences and citations with their amplification success, per hemipteran family, in this study. Primer sequences are provided with their first occurrence, and sequences for cocktail primer components are provided below. All cocktail primers are used in a 1:1 ratio.

Most of specimens in this study were processed using high-throughput protocols at the Canadian Center for DNA Barcoding (CCDB), available at: http://www.ccdb.ca/resources.php. These techniques are an integrated workflow of specimen preparation, data recording, photography, tissue sampling, sequencing, and data integration managed using a laboratory information system (LIMS)—which results in individual specimen pages, connected through BINs on BOLD [1,8,18,19,20,21]. The full laboratory history for each specimen can be accessed via its sequence page link on BOLD, with the exception of a few specimens that were processed in other facilities. Fig 1 details the primers used to generate sequences included in the data release, whereas S2 Table lists the sources/collectors of all data.

**Fig 2:**
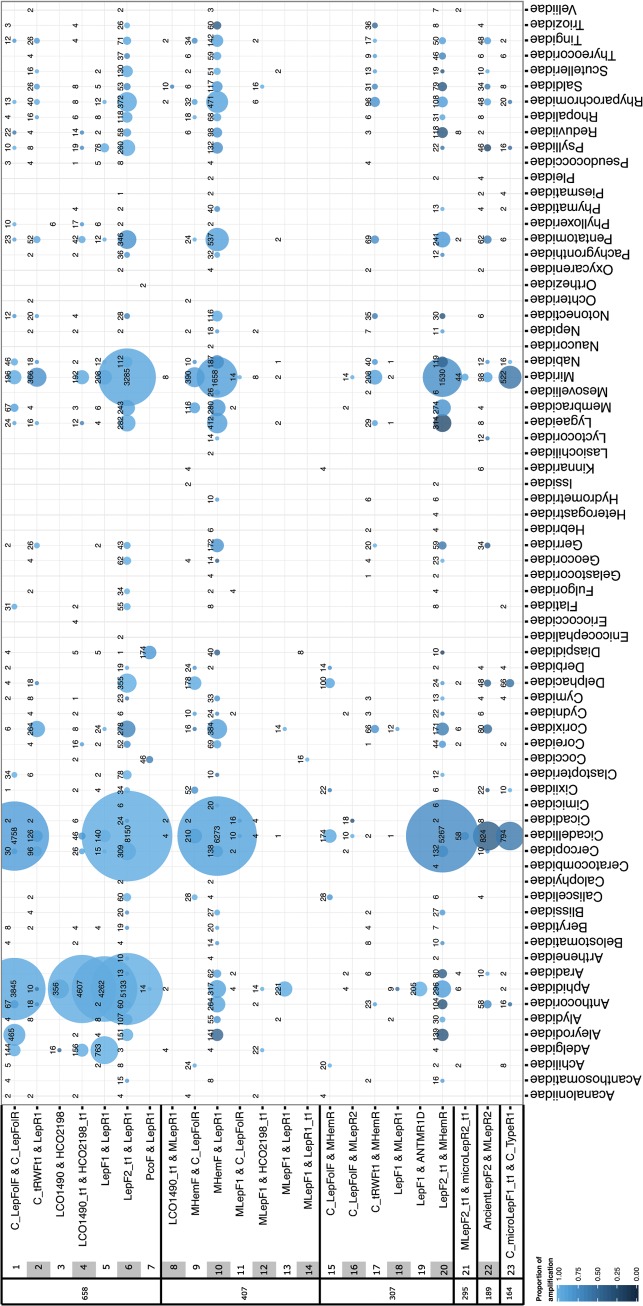
Proportions of PCR amplifications by taxon, generated with particular primer pairs, for all Hemiptera specimens in this study. This heatmap pairs with Fig 1, where the sequentially shaded numbers in the left columns connect primer sequences and citations with their use, per hemipteran family. Primer pairs appear in descending order of amplicon lengths, at left. The dot area is proportional to all amplification attempts for that primer/family, the total number of specimens analyzed per primer combination is adjacent to each dot, and the shading indicates the proportion of successful amplifications (illustrated in the key at lower left). All cocktail primers are used in a 1:1 ratio.

### Choice of a Taxonomic Checklist/Catalogue

The “Checklist of the Hemiptera of Canada and Alaska” by Maw et al. [17] was used as the sole nomenclatural basis for this study, although several more recent studies have led to some shifts in generic and species revision [22,23,24] (also, G. G. E. Scudder (University of British Columbia, CA), C. H. Dietrich (University of Illinois Natural History Survey, USA), C. Bartlett (University of Delaware, USA), J. N. Zahniser (University of Illinois Natural History Survey, USA), personal communication). Generally, all but the most recent catalogues require revision, and the adoption of a particular list/catalogue (or set) during the initial library construction provides a nomenclatural baseline against which future updates can be compared. Here, all new taxonomic identifications for this study were made consistent with the names of Maw et al. [17].

### Specimen Collection and Processing

Specimens in this study originate from museum collections, from recent contemporary mass-collecting efforts, and from colleagues contributing public records on BOLD. Museum specimens were borrowed under a formal Memorandum of Understanding with the source institution (see institutions listed in S3 Table). When borrowing museum specimens for DNA Barcoding [25,26], we used the workflows given in Hebert et al. [21, see for descriptive photos] described in brief here. Tissue sampling from museum specimens involved the removal of a mid, or hind leg; and for some recent collections or very small specimens, such as those from malaise traps, tissue sampling involved whole specimens preserved as vouchers. High-throughput laboratory protocols are based on a 96-well plate format, and specimens are collected into (and returned in) customized Schmitt boxes [27] arrayed into 96 plate positions. As specimens were removed from the collection, a unique CCDB label was swapped into the specimen’s location, indicating the specimens’ unique Schmitt box and CCDB accession number. Wherever possible, we selected 3–5 specimens of each species that was already represented in BOLD, targeting those with clear locality data, sex determination, their membership in a type series, and from a series where the specimen’s labels indicated it was part of a prior study. Small insect specimens, especially those 20 years old or older have lower sequence recovery rates [28] but this can be compensated for by sampling multiple individuals [21]. In this study, the youngest specimens, approximating the above criteria, were preferred.

Because recently collected specimens can yield high-quality sequence results, we supplemented work on museum collections by analyzing hemipterans collected by a range of methods, including Malaise traps, sweep netting, and pitfall taps. These methods include the School Malaise trap program [29] (an array of Malaise traps deployed across southern Ontario) and the BioBus [30], a technician-run collection vehicle specializing in continent-scale specimen collection for DNA barcoding. No specific collection permissions were required for recently collected specimens in this study, as they do not involve endangered or protected species and were previously collected for other projects under blanket collecting permits and permissions issued to the Biodiversity Institute of Ontario. Specimen details, including the holding institution, and original accession number, are provided with each specimen’s record on BOLD, and are accessible through the DOIs and project names mentioned above (see **Data Release).**

When initially compared against identified North American Hemiptera on BOLD, these recent collections (primarily collected in 2012–2013) yielded over 1000 BINs not identified to species. We prioritized the identification of 2–3 specimens from a selection of these BINs based on collection location, as well as those that most closely matched a species on the Canadian checklist, when queried on BOLD, using its ID Engine [1]. This subset of specimens was identified by taxonomic specialists, whose working time was greatly reduced by preliminary identification via BOLD [31] [also, C. Bartlett (University of Delaware, USA), M. D. Schwartz (Canadian National Collection of Insects, Arachnids and Nematodes, CA), J. N. Zahniser (University of Illinois Natural History Survey, USA), personal communication]. When specimens of species were unavailable for Canadian localities, we sought representatives from the US or Mexico.

### Specimen Identification: how specimens on BOLD get their names

BOLD is a wiki-like environment [32], and all taxonomic names are supplied by data-submitters (i.e., those with edit-level access to particular records). Fields are also available to indicate the identifier and the identification method, and we encourage all data-submitters to clearly indicate their identification method. Records in BOLD obtain their taxonomic information from any of three sources, described below.

1) User submitted names: data-submitters indicate species names, and other taxonomic information during data submission; this includes identifiers indicated on museum specimen labels.

2) Institutional names: Insect specimens, in museum collections, are often assigned to a species’ unit tray by curators, or visiting specialists. When these specimens do not have a determination label, we listed the identifier as [Institutional] Curator with the name, or institutional abbreviation (e.g., Smithsonian Curator, or CNC curator), and the identification method is “Institutional ID”.

3) BOLD ID Engine, or BIN-based match names: The BOLD ID engine first aligns a protein translation of the query COI sequence, using a Hidden Markov Model, to a ‘query-optimized’ alignment-set of specimens on BOLD [1]; the composition of this set corresponds to the search database chosen by the user, during the query. The engine then performs a linear search of this ‘query-optimized’ set to produce a match. The BIN algorithm (mentioned above) aggregates unidentified and identified specimens together in a BIN. In brief, the BIN algorithm does this by a staged clustering process using uncorrected pairwise distances. The process uses a threshold distance for an initial clustering step, and further refines groupings within, and between these clusters via Markov Clustering [8]. This is a dynamic process that depends on the data available, and for a detailed explanation of this process, please sees pages 2–6 by Ratnasingham & Hebert [8]. These algorithms provide a suggested identification, but the taxonomic names of specimens can only be changed by the data owner through submitting an update to BOLD.

### Data Analyses

The main workflow for analyses, tables, and figures was written and performed in R [33] using a BOLD-formatted download (as a csv file) as input (this was all data from BOLD projects: *Hemiptera of Canada—Main dataset*, *parts I and II*, in DOIs dx.doi.org/10.5883/DS-HECAMAIN, and dx.doi.org/10.5883/DS-HECAMN1). The analyses can be reproduced using the annotated R file, and can be adapted to accommodate other taxa. R code and all supporting R workflow data files are available as S1 Code and described in the legend for S1 Code. R code in-development is deposited at https://github.com/RodgerG/Hemiptera-of-Canada-DNA-Barcode-Library-workflow.git, and the version used for this study (V1.0) is at the DOI: http://dx.doi.org/10.5281/zenodo.12582. For all analyses involving the library dataset, we use the set available as BOLD project: *Hemiptera of Canada—DNA barcode library* at DOI dx.doi.org/10.5883/DS-HECALIB.

Specimens collected in Canada, in BINs without any named specimens, represent possible new species records on BOLD. These records are part of the data release, but not included in the library dataset.

To investigate the utility of library DNA barcodes to differentiate species, we calculated the number of species per BIN, from the library. BINs containing a single species are considered to be concordant; those with more than one species are considered discordant. Concordance will vary between the library and on BOLD because the number of records in need of editing or revision for various taxonomic, or metadata-quality reasons, will be higher on BOLD; and these records tend to influence a species’ status toward discordance.

Library species that could be successfully identified were scored as those only occurring in concordant library BINs (BINs containing only one species name). Species sharing barcodes (species that have specimens in discordant library BINs) were determined with the same analysis. This analysis determines species’ concordance only within the library dataset. To determine a library species’ concordance on BOLD, all library specimens were also analyzed via a BIN discordance report (an online tool) on BOLD. For all library species concordant on BOLD (a conservative estimate), intraspecific divergence was calculated with a pairwise distance analysis using the Kimura 2-parameter model [43] as implemented in BOLD, and the mean standard error, minimum and maximum intraspecific sequence divergence was calculated in R. Potential cryptic species, with mean sequence diversity >2%, were identified with the same analysis.

To provide an overview of the library’s contents, we visually explored several aspects of the data. BIN discordance among specimens from the three largest taxonomic families (Aphididae, Cicadellidae, Miridae), identified by taxonomic specialists, was calculated using the same method for barcode sharing (above), and visualized in three dimensions: by family, by number of species in a BIN, and by number of specimens in a BIN. Also, primer usage and proportion of amplification success for all specimens in the data release was plotted as a proportional heatmap. The primer usage data were provided by the BOLD technical staff.

## Results

### Overall Diversity

The Checklist of Hemiptera of Canada [16] recognizes 78 hemipteran families, 870 genera, and 3944 species as native, or naturalized to Canada. The general data release of specimens matching this checklist or unidentified from Canadian localities, is in the BOLD projects: *Hemiptera of Canada—Main dataset*, *parts I and II*: available at the DOIs dx.doi.org/10.5883/DS-HECAMAIN, and dx.doi.org/10.5883/DS-HECAMN1 and contains 54,280 records, all identified to family (74 families), 39,493 identified to genus (769 genera), and 28,408 identified to species (2,671 species). All records comprise 2,714 BINs, of which 736 are unnamed; specimens from unnamed BINs are presented in S4 Table.

Among samples borrowed from museum collections, we find at least 94 species from Canadian localities not recognized by Maw et al., representing a potential ~ 3% increase of the known fauna. The workflow, after processing through the API of the Catalogue of Life (COL) [34], initially identified >280 species as tentatively new to the checklist. Further nomenclatural review revealed many ‘new’ species to be: previously published synonyms (101 species), species described or reported since Maw et al. 2000 (49 species), spelling or case agreement errors (29 species), or require further taxonomic clarification (>30 species). Brief status notes for the remaining species tentatively considered new to the checklist are presented in S5 Table, and the specimens are available in the BOLD project *Hemiptera of Canada—Tentative New Specimen/Species Records*, via DOI dx.doi.org/10.5883/DS-HECATEN.

**Fig 3:**
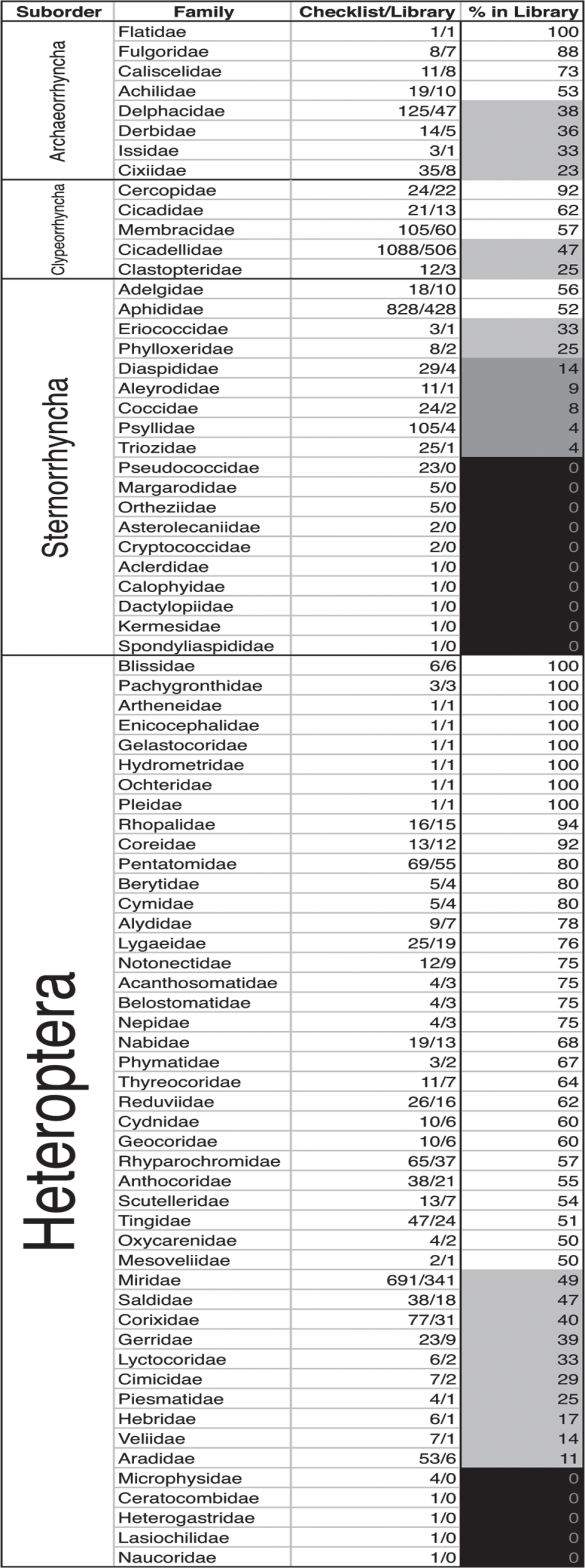
Species-level summary by family, of library coverage given the Checklist. Families are grouped by suborder, and then by the proportion of species with barcode records. The numbers in the Checklist/library column indicate the number of species for each family in the Checklist, and the number with barcode coverage.

We present a DNA barcode library for the Hemiptera of Canada containing 20,851 specimens classified in 64 families, 628 genera, and 1849 species, assigned to 1867 BINs that can be accessed at the DOI dx.doi.org/10.5883/DS-HECALIB; instructions for specimen-level access are provided as S1 Instructions. A summary table of the taxonomic contents at the ordinal level for the data-release and library are presented in Table 1. A summary of species-based library coverage at the family level is presented in Fig 3. The species-level diversity for library specimens is presented as an annotated version of the Maw et al. checklist in S6 Table. Annotation for each species in S6 Table includes nomenclature status on the Catalogue of Life [34], specimen numbers, divergence metrics, barcode-sharing, and BIN discordance both within the library, and on BOLD. We find 1,312 library species correspond to concordant BINS—within—the library (368 of these species are represented by singleton BINs); 510 Library species appear to share BINs (are discordant within the library), and 27 have not yet been placed in a BIN (as of this writing); species-specific results are presented in S6 Table. Of the 1,312 library species in concordant or singleton BINs, 919 can be successfully identified based on their concordance on BOLD (741 species are in concordant BINs on BOLD, and 178 are singleton BINs; S6 Table). Lastly, we find 27 library species with > 2% mean intra-specific pairwise divergence, which may represent cryptic species complexes, listed in S7 Table.

**Table 1:** Ordinal-level summary for all data-release and library specimens.

**Suborder**	**Lib.Families**	**Families**	**Prop.Named.Family**	**Lib.Genera**	**Genera**	**Prop.Named.Genera**
Not.assigned	1	3	0	1	3	0
Archaeorrhyncha	8	8	1	35	47	0.73
Clypeorrhyncha	5	5	1	146	186	0.97
Heteroptera	41	46	1	291	358	0.98
Sternorrhyncha	9	12	1	155	175	0.87
**Totals**	64	74	*	628	769	*
**Suborder**	**Lib.Species**	**Species**	**Lib.BINs**	**total.BINs**	**UnNamed.BINs**	
Not.assigned	2	3	5	157	129	
Archaeorrhyncha	87	177	83	119	20	
Clypeorrhyncha	604	875	594	867	227	
Heteroptera	703	1135	724	822	76	
Sternorrhyncha	453	481	461	749	284	
**Totals**	1849	2671	1867	2714	736	
**Suborder**	**Lib.>2%div**	**Lib.Specimens**	**Specimens**	**Prop.Named.Specimens**	**Lib.spp.sharing.barcodes**	
Not.assigned	1	8	10117	0	0	
Archaeorrhyncha	0	527	1222	0.63	23	
Clypeorrhyncha	11	7373	15806	0.6	225	
Heteroptera	12	4724	10823	0.85	166	
Sternorrhyncha	2	8219	14587	0.61	96	
**Totals**	26	20851	52555	*	510	

Shaded columns indicate library specimens, light columns indicate those from the data-release. This table provides a suborder level totals of specimens by taxon-based coverage, proportion of named specimens, BIN totals, species sharing barcodes, and those with >2% divergence. Specimens not assigned to family represent specimens with nomenclature-yet-to-be-confirmed, or specimens without DNA barcodes. The “Not Assigned” category is a workflow-filter that identifies specimens with species names consistent with the checklist, but whose higher taxonomy is not consistent with the checklist.

### Visual Data Summaries

**Fig 4:**
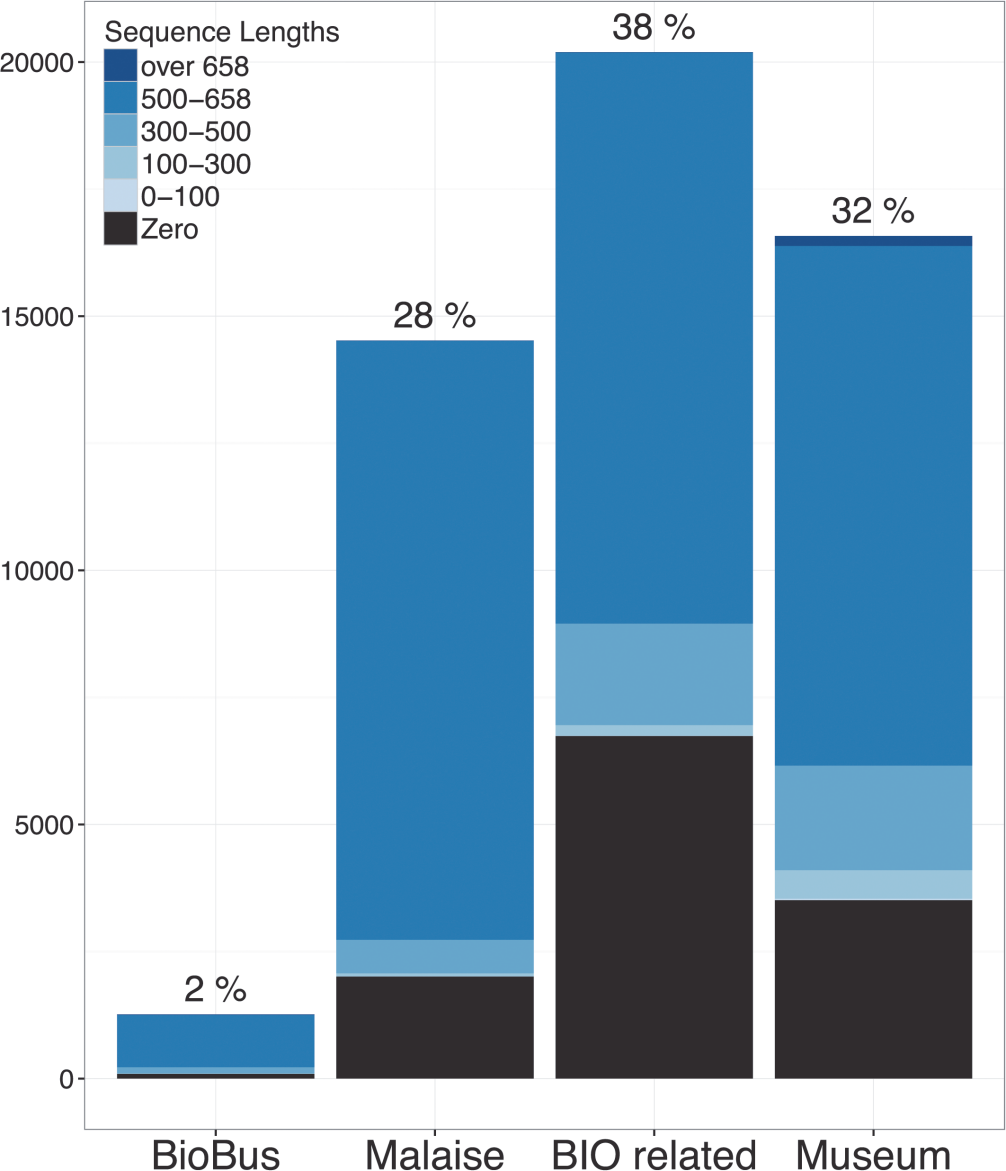
Proportion of data sources, and sequence length for all records in this study. These generalized-collection methods acquire a broad spectrum of taxa, and are valuable sources of diversity when combined with conventional museum collections, and research projects through BIO. Some specimens, especially those from museums, lack sequence data, but do have images, names, and associated metadata on BOLD.

Specimens in the data-release come from several sources (Fig 4), including sampling of species from Institutional collections and a range of fresh collection methods. The number of specimens representing species in the library is a function of taxon abundance and representation in collections, and the rate of successful DNA barcode sequence recovery.

Although most of the available sequences are from are recently collected specimens collected within the past few years (S1 Fig), the majority of specimens accessioned with names are older specimens from museums. Less than 30% of all data-release specimens come from museums, but museums account for ~ 50% of the library specimens (S2 Fig). This disparity highlights both the importance of museum specimens to provide a context for specimens from recent projects, and the utility of the latter for extending coverage of geographic range and COI sequence variability within and among taxa.

The number of species per family in the data-release (Table 1) and library (Table 1 and Fig 3) reflects the relative diversity of those taxa in the checklist (S6 Table). This representation is not only frequency-dependent, reflecting fresh captures from recent projects (Fig 4), but also corresponds to contributor interest, as the more species-rich groups contain members of economic or systematic importance (in particular: the Aphididae, Cicadellidae, and Miridae; Fig 2 and Fig 3). However some relatively diverse groups have limited representation, notably scale insects (Coccoidea) and jumping plant lice (Psylloidea). These groups not only require specialized collecting, preservation, and identification techniques, but also museums do not usually maintain specimens of some groups in a state usable for DNA Barcoding (e.g., specimens are slide-mounted or preserved in inappropriate fluids).

Amplification success by primer (Figs 1 & 2) tends to be highest with full-length (658bp) DNA barcode primers (Fig 2) applied to specimens collected within the past 30 years (S1 Fig). Particularly, shorter-fragment “mini” primers (Figs 1 & 2) have mixed success across many groups, and these primers are often attempted with older or museum specimens after full-length DNA barcode primers are unsuccessful. Amplification bias does not appear to be systematic across families, but in some cases may be taxon-dependent (e.g., primer pairs applied within the Aleyrodidae, Corixidae, or Miridae), and Fig 2 may assist targeting amplification by taxon.

**Fig 5:**
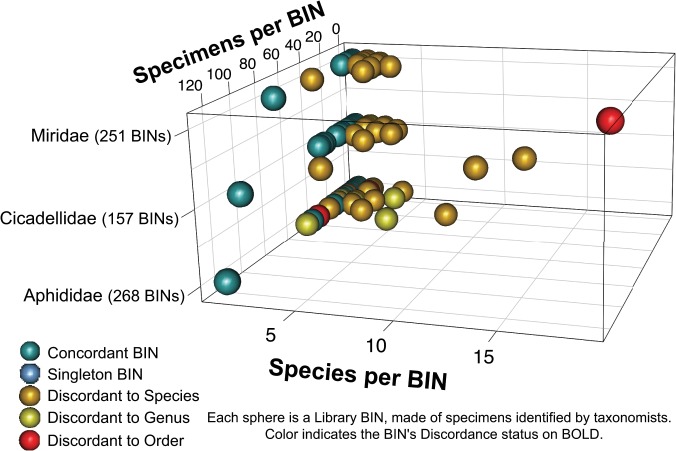
A three dimensional visualization of taxonomic congruence between BINs and species identifications, based on morphology, for three families of Canadian Hemiptera (Aphididae, Cicadellidae, and Miridae). The taxonomists who identified these specimens are listed in S7 Table. A robustly concordant BIN (one species per BIN, with many specimens in that BIN) occupies a forward position in the plane along the left wall, whereas discordant BINs (those with many specimens, and several species names) appear towards the upper right quadrant. The colors correspond to the concordance status of the same BIN on BOLD. In many cases, BINs that are concordant in this curated dataset are discordant on BOLD. This disparity highlights the utility of defining a reference set of specimens, as library specimens will be grouped by BOLD in BINs with misidentified specimens.

BIN discordance among library specimens identified to species by taxonomists is presented in Fig 5. This figure displays a range of congruent and incongruent states that BINs for a taxon, or entire library can possess, based on the diversity of specimen information, and DNA barcode variation aggregated in an individual BIN. A table of these taxonomists and their number of specimens is provided in S8 Table. Of the 676 BINs in Fig 5, the majority (606 BINs) are concordant (contain one species), 45 BINs have two species, and 25 have three or more. An expanded view of BIN discordance for all families in the library is presented in S3 Fig, and the taxonomists involved are listed in S9 Table.

## Discussion

We have established a DNA barcode library with reference to a taxonomic list or catalogue for an order of insects within a country, using a DOI to define library specimens on BOLD. Additionally, we have made the R-based workflow publically available, and reproducible through the S1 Code. This library presents a defined set of reference specimens of the Hemiptera of Canada, which is open to community review and comments (to do this, please see S1 Instructions). Using the data from a DOI in an R-based workflow, several new developments are possible. We found the ability to rapidly categorize a burgeoning stream of BOLD data against a taxonomic catalogue, and the existing library allowed us to dynamically identify new library specimens, as well as tentative new species records, and unnamed BINs both of which were subsequently identified by taxonomic review. Automatically querying taxonomic names of new records against the Catalogue of Life using ‘taxize’ [35] easily revealed nomenclatural differences between ‘new’ records, and our catalogue with the most comprehensive global index available [34]. These ‘new’ species records (initially >280 species) were obtained through general museum surveys, similar to the methods of Hebert et al. (2013) [21]—and we hypothesize this result may apply for most taxa. Manual research revealed that many of these ‘new’ records (S5 Table) actually occur in the checklist, because our auto-processing missed them due to: synonymy, changes in generic combination, misspelling/wrong gender agreement from both BOLD and/or the checklist, and differences in recognition/non-recognition of subspecies. Lastly, because the R-workflow is based on a BOLD-formatted spreadsheet (the standard data download from BOLD), it is adaptable for most BOLD users, and most datasets.

The past several years have seen a rise in Hemiptera DNA barcode publications that span intra-specific [36,37] and inter-specific [38] population studies, regional libraries from Europe [39], India [40], Korea and the far east [41], and the start of a global focus at the family (Aradidae) [42], and suborder (heteroptera)[43] levels. Thus far all Hemiptera libraries, including this study, predictably find patterns of taxonomic agreement, cryptic diversity or barcode sharing based on their specimens examined. These preliminary observations are important, but isolated, in light of the comparative contributions each study can provide, if regional, or taxon-based libraries could be collaboratively organized on a global scale.

Prior to developing our workflow we considered 41 citations, spanning the past five years, which explicitly discuss creating a DNA barcode Reference library (presented in S1 Table, which includes search methods, and keywords). Approximately half of these 41 studies proceed with an a priori taxonomic catalogue, whereas the others generate a post-hoc list from the study results. Very few are continental (3 studies) or global (1 study) in scope, and there is a clear taxon bias toward insects (19 studies), toward fish among vertebrates (7/10 studies) and a pioneering representation for community assemblages. While data from most of these studies is publically available, we found none combined a prior taxonomic hypothesis (catalogue) with a publically available dataset designated by a DOI, and developed a scalable workflow relative to their data-sources. Development of an open-source workflow that is reproducible serves as a common-source tool for broad community use [44]. Such community ‘conversation’ is a frontier in cybertaxonomy [45], and we offer our workflow as a point for discussions toward other library construction efforts. BIN discordance exists in the library at various taxonomic levels (Fig 5 and S3 Fig), and one of the next developmental steps for the Hemiptera of Canada library is specimen-level annotation by taxonomic specialists. Similar to the curation of museum-specimens by external experts, such annotations could accumulate a community consensus of comments on particular specimens (or BINs) (please see S1 Instructions for a step-by-step guide to accessing and annotating library specimens, or BINs).

Currently, queries on BOLD are confined to the OpenIdEngine (http://www.boldsystems.org/index.php/IDS_OpenIdEngine), and direct queries via BOLD of the library we present here, are not yet available. However, library specimens can be added to any private project, and then used with all of the analysis tools available on BOLD. Furthermore, the library contents can be accessed directly (see S1 Instructions), and the ProcessIDs/SampleIDs from library specimens can be compared to search results from the OpenIdEngine, or to specimens in BINs.

The library contents range from species represented by a single specimen to concordant species spanning several BINs with hundreds of specimens per BIN; and although this first draft of the library varies in taxonomic coverage and agreement, several patterns useful for making identifications are becoming clear. Many species represented by singleton BINs (and thus, a single specimen), such as the mirid plant bug *Tropidosteptes cardinalis* (Uhler, 1878)[46] (http://www.boldsystems.org/index.php/Public_BarcodeCluster?clusteruri=BOLD:AAG8871), are important first-references that can be prioritized for further sampling in future library drafts. Some species with concordant BINs have very few specimens identified by a taxonomist, relative to, perhaps, hundreds of specimens collected from across the continent (for example, the cicadellid leafhopper *Macrosteles quadrilineatus* (Forbes, 1885)[47] (http://www.boldsystems.org/index.php/Public_BarcodeCluster?clusteruri=BOLD:AAA9422), and these BINs appear to reflect a clear biological grouping.

BIN discordances within the library (e.g. Fig 5) are likely to be biological realities, and may also highlight inconsistencies in identifications or application of species concepts by different workers. Some taxonomic groups vary widely in their concordance, for example the plant bug genus *Lygus* (family Miridae). A single BIN corresponds to *Lygus lineolaris* (Palisot, 1818)[48] that contains > 700 specimens collected from across North America, with identifications by multiple taxonomists (http://www.boldsystems.org/index.php/Public_BarcodeCluster?clusteruri=BOLD:AAA5803).

However, another *Lygus* (Hahn, 1833)[49] BIN includes ~ 600 specimens of 27 different taxonomist-identified morphospecies (http://www.boldsystems.org/index.php/Public_BarcodeCluster?clusteruri=BOLD:ACF4388), reflecting an intriguing taxonomic issue (G. G. E. Scudder & M. D. Schwartz, personal communication from identifiers of these same *Lygus* specimens). Lastly, even discordant multi-species BINs represent a useful level of identification when patterns of discordance are consistent, especially in genera containing one or more complexes of closely related species.

### Future Directions

Comparison of additional specimens with this first iteration of a DNA barcode library of the Hemiptera of Canada is a means of identifying candidate species that can contribute to the development of a more complete dataset [50,51,52]. As additional data become available on BOLD, and the Hemiptera of Canada library grows, targetable gaps in taxon and geographic coverage become clear—clarifying the contributions needed from taxon specialists, and highlighting species of interest that can be targeted by novel resources, such as DNA barcode Bioblitzes [53], or student projects via the Education Barcode of life’s (eBol) student data portal [54].

Lastly, we recognize the multimodal nature of biodiversity inference can be unifying. DNA sequences without names, specimens without sequences, names that are synonymies—can all be reciprocally illuminating in a comparative framework. Conventionally, these frameworks have been important, but isolated works: catalogues, monographs, revisions, descriptions, sequences, and their epistemological foundation—specimens. As demonstrated here, an infrastructure that digitally aggregates specimens in a DNA-mediated reference library can unite these isolated resources into open-sourced ‘virtual unit trays’ of specimens, and sequences, from across the world’s collections. Such an effort integrates isolated resources to create a shared understanding about taxon diversity.

Communicating about how we evolve such an infrastructure is a frontier in biodiversity science. And we share the ideas in this paper to help evolve inference methods that are at once public, repeatable, collaborative, and comparative.

## Supporting Information

S1 Fig Specimen number and sequence length recovery by year for specimens included in the data-release. Years are grouped by decade until 2000–2005 to reflect the recent rise in number of specimens added to BOLD in each year.
(EPS)

S2 Fig Proportion of data sources, and sequence length for the records included in the library dataset, showing museums as an important source for library specimens. (EPS)

S3 Fig A three dimensional visualization of taxonomic congruence between BINs and species identifications based on morphology, for all families of Canadian Hemiptera; this presents a snapshot of the contents and structure of the entire ‘library’. Such a view could be used to access macro-level progress during early phases of library construction. The taxonomists who identified these specimens are listed in S9 Table. A robustly concordant BIN (one species per BIN, with many specimens in that BIN) occupies a forward position in the plane along the left wall, whereas discordant BINs (those with many specimens, and several species names) appear towards the upper right quadrant. The colors correspond to the concordance status of the same BIN on BOLD. In many cases, BINs that are concordant in this curated dataset are discordant on BOLD. This disparity highlights the utility of defining a reference set of specimens, as library specimens will be grouped by BOLD in BINs with misidentified specimens.
(EPS)

S1 Instructions Step-by-step instructions for accessing and commenting on individual records within the DNA barcode library for the Hemiptera of Canada. These same instructions are applicable for adding comments to a particular BIN page.
(PDF)

S1 Code Zipped file containing data, accessory files and R code to reproduce all analyses and tables presented in this paper. In R, once the working directory is set to a folder containing these files, all code can be executed and run at once. The R code calls for the installation and use of all necessary packages although mac users may need to update their installation of XQuartz (http://xquartz.macosforge.org), to render the three dimensional images presented in Fig 5, and S3 Fig. For further details, please see the annotation in the preamble, and throughout the R code.
(ZIP)

S1 Table Library citations and brief characterization of the methods employed by publications describing the construction of DNA barcode libraries. Construction-method categories were arbitrarily designated after considering the diversity of methods used in all publications.
(PDF)

S2 Table Contributors of specimens for all data released as part of this manuscript. These data can be found on BOLD, in projects: ‘*Hemiptera of Canada—Main dataset parts I and II’* that can be accessed via two DOIs: 1) dx.doi.org/10.5883/DS-HECAMAIN; 2) dx.doi.org/10.5883/DS-HECAMN1.
(CSV)

S3 Table Institutions that provided specimens for processing at the Canadian Center for DNA Barcoding. (CSV)

S4 Table Specimens in BINs that lack specimen with a species assignment. These specimens are found, using R, by identifying all BINs containing species, and excluding all specimens in these BINs, through a subset of the data. These taxa represent tentative new species records for the DNA barcode library, but they await identification.
(CSV)

S5 Table Tentative new species records for Canada. These specimens are found, using R, by identifying species that do not match the Maw et al. (2000) checklist, but that were collected within Canada. Duplicate entries have been removed. These specimens derive from thorough sampling of museum collections and from targeted searches by taxon. Many of these species have been recognized as native or naturalized after Maw et al (2000), and each species has been annotated with brief notes regarding its tentative taxonomic status. The full list of specimens is available on BOLD in the project *Hemiptera of Canada—Tentative New Specimen/Species Records*, available at the DOI dx.doi.org/10.5883/DS-HECATEN.
(CSV)

S6 Table Species-specific information for taxa included in the Maw et al. (2000) checklist, as found in the reference library, and on BOLD. The curated dataset of the library contains fewer specimens, and fewer cases of BIN discordance than the full public data on BOLD, and this information is summarized here. For each species, this table also lists the taxonomic status of each on the Catalogue of Life, the number of specimens on BOLD, the number of specimens in the library, the mean, standard error, maximum and minimum intraspecific pairwise divergence (using the Kimura two-parameter model available on BOLD), whether a species shares barcodes and, if so, the number of species involved, the number of BINs per species, and lastly the number of specimens in BINs on BOLD, relative to the BIN’s concordance status.
(CSV)

S7 Table Species in the library dataset that display greater than 2 percent uncorrected pairwise divergence. It is possible these species represent either particularly divergent, or cryptic groups.
(CSV)

S8 Table Taxonomists whose identifications were used to generate the concordance reports in Fig 5. (CSV)

S9 Table Taxonomists whose identifications were used to generate concordance reports for S3 Fig. (CSV)
