# Supplementary material for: The Hemiptera (Insecta) of Canada: Constructing a Reference Library of DNA Barcodes
Source: PLoS One. 2015 Apr 29;10(4):e0125635. doi: 10.1371/journal.pone.0125635 (PMC4414572; doi:10.1371/journal.pone.0125635)
Supplement: S1 Instructions — These same instructions are applicable for adding comments to a particular BIN page. (PDF) [file pone.0125635.s004.pdf]

The instructions below are for searching and accessing Hemiptera of Canada Library Records on BOLD. Here we will retrieve a specimen record for a seed bug *Northolomus scolopax*, collected and identified by noted Canadian Heteroperist Geoffrey G.E. Scudder.

Please create a BOLD account, and login at http://www.boldsystems.org/

Once logged in:

1) Choose "Workbench" (at top of page)

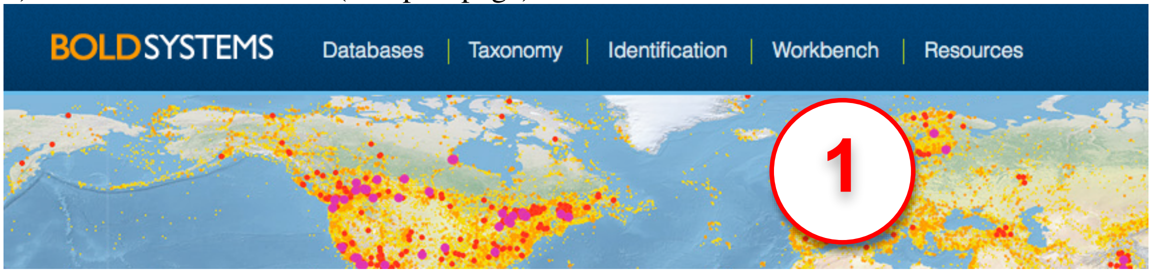

2) Open "Public Datasets" (at center left of page)

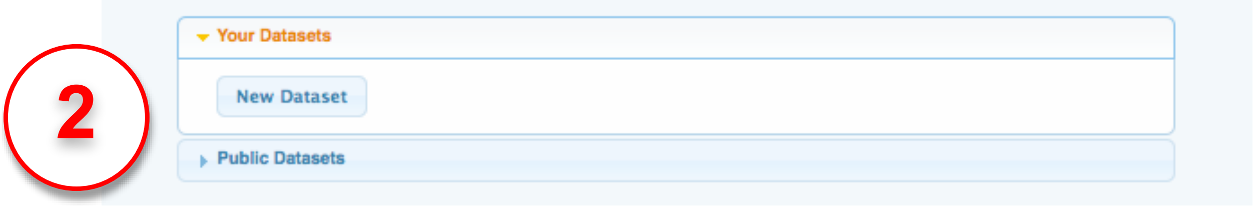

3) Click "Rodger Gwiazdowski" (alphabetically listed by first name)

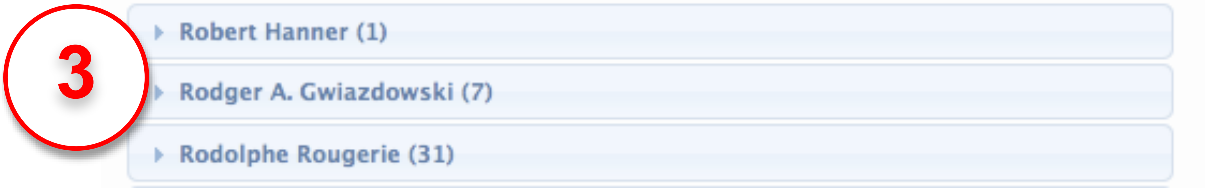

4) Click on the dataset ID: "DS-HECALIB" for the Hemiptera of Canada Library

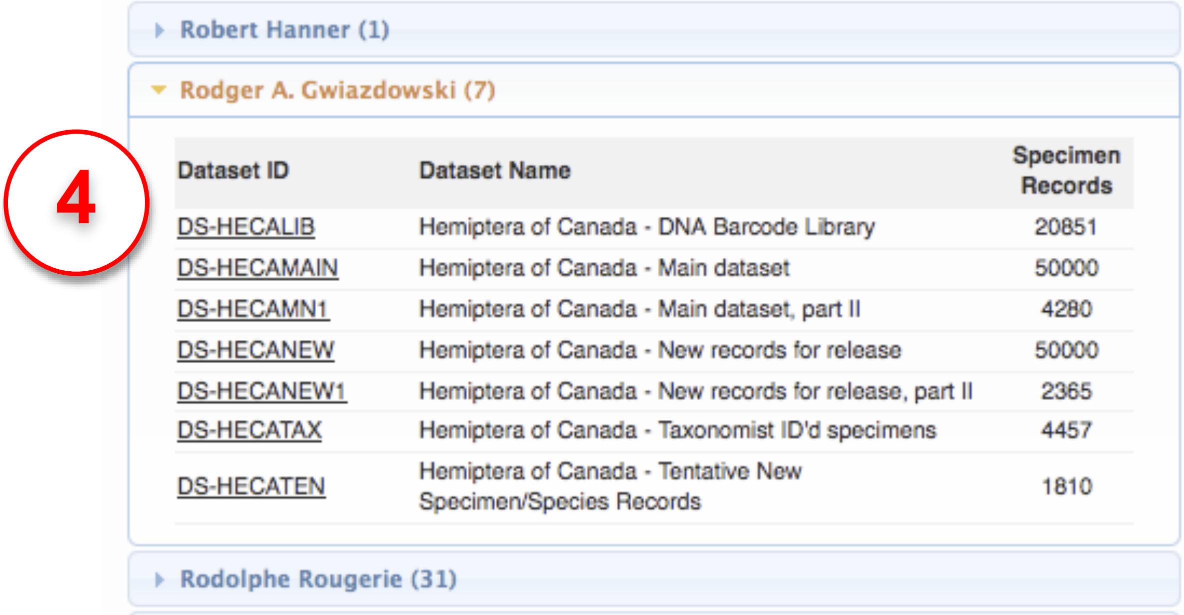

Dataset ID	Dataset Name	Specimen Records
DS-HECALIB	Hemiptera of Canada - DNA Barcode Library	20851
DS-HECAMAIN	Hemiptera of Canada - Main dataset	50000
DS-HECAMN1	Hemiptera of Canada - Main dataset, part II	4280
DS-HECANEW	Hemiptera of Canada - New records for release	50000
DS-HECANEW1	Hemiptera of Canada - New records for release, part II	2365
DS-HECATAX	Hemiptera of Canada - Taxonomist ID'd specimens	4457
DS-HECATEN	Hemiptera of Canada - Tentative New Specimen/Species Records	1810

5) You are now on the Dataset Console, from which you can access all records by:

A) Viewing all records

B) Downloading all library data as a spreadsheet

C) Searching by taxonomy (geography, or other metadata); see details below.

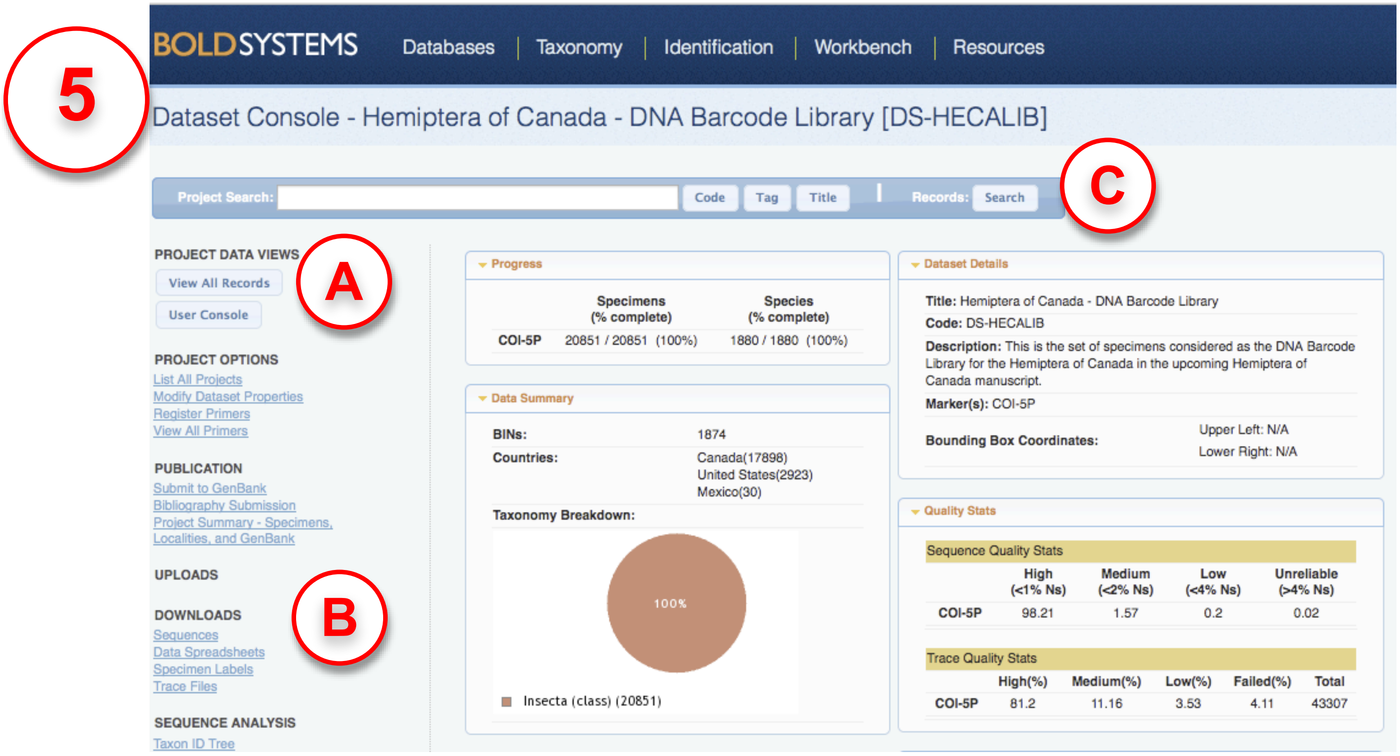

6) On the Record Search page you can search for records in several ways.
To search by taxonomy: click "Search" (C), (for the record search window) entering search terms as "Genus Species" will find that species. Note that you need the include the quotes " ", around the binomial name, otherwise the search engine will return all results for that genus species epithet.
*Note that Record Searches, that search within a dataset [e.g.: DS-HECALIB] occur by default, when engaging a search within that dataset.

Directly access specimen records using the Process ID (and ID number given during the CCDB laboratory processes) Sample ID (an ID number given during the specimen's accession to BOLD) or BIN number from the Record Search window:
Click the "List of Identifiers" tab (adjacent to the "Region of Map" tab), enter (type/paste) the record number (s) in the window and click "Search Records".
Search using either:
Process ID: CNCHA224-11
Sample ID: CNC#HEM300524
* Many Process IDs/Sample IDs can be searched at once.

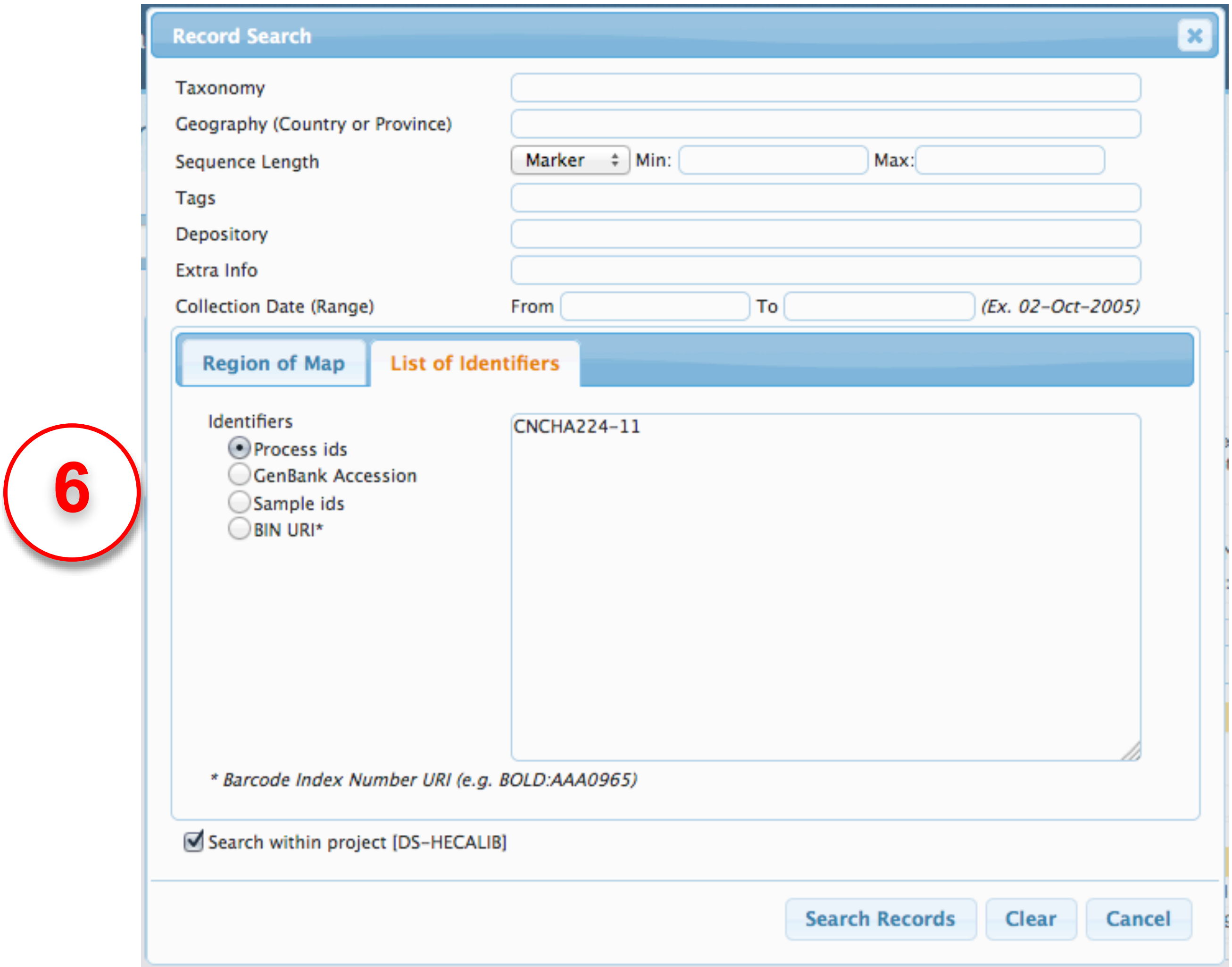
6
Record Search
Taxonomy
Geography (Country or Province)
Sequence Length
Marker
Min:
Max:
Tags
Depository
Extra Info
Collection Date (Range)
From
To
(Ex. 02-Oct-2005)
Region of Map
List of Identifiers
Identifiers
Process ids
GenBank Accession
Sample ids
BIN URI*
CNCHA224-11
* Barcode Index Number URI (e.g. BOLD:AAA0965)
Search within project [DS-HECALIB]
Search Records
Clear
Cancel

7) Commenting on the identification, or any other information on a Library record is welcomed! . Currently, there are two ways anyone can add information to records:

1) Direct Annotations: each specimen page, and BIN page has a button for public annotation via posted comments (D).

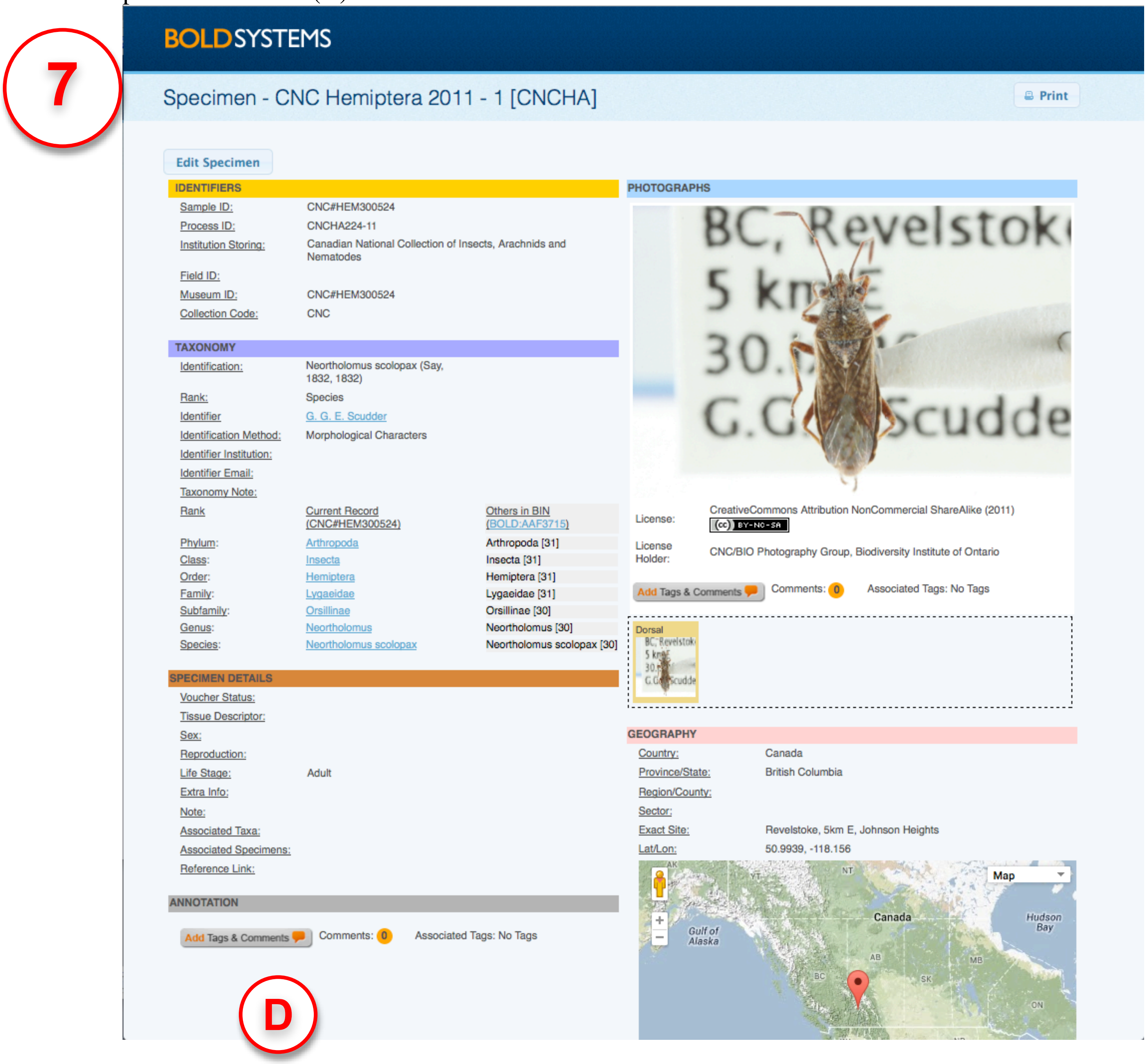

2) Editorial Access: editing access for public records can be obtained from the BOLD technical staff. We invite those interested to edit records to please contact the authors, or the BOLD team at support@boldsystems.org with the nature of your request or observation, and to please include specimen Process IDs (or Sample IDs) where applicable.
