# Supplementary material for: The Hemiptera (Insecta) of Canada: Constructing a Reference Library of DNA Barcodes
Source: PLoS One. 2015 Apr 29;10(4):e0125635. doi: 10.1371/journal.pone.0125635 (PMC4414572; doi:10.1371/journal.pone.0125635)
Supplement: S1 Table — Construction-method categories were arbitrarily designated after considering the diversity of methods used in all publications. (PDF) [file pone.0125635.s006.pdf]

Supplementary Table 1.

Review of Prior DNA Barcode Library Construction Methods:

To explore construction methods for studies that suggest, or indicate creation of a DNA Barcode library, we performed a literature search via a Web of science "Topic Search" (periodically, from October 2013 - March 2014) using the keywords:

DNA Barcode Librar*
DNA Reference librar*
Barcode reference librar*
Barcode librar*
Barcode ref*.

For each publication we subjectively evaluated the scope, taxa involved, and categorized each work into general patterns of construction.

Results:

We find 41 citations, spanning the past five years that explicitly discuss creation of a DNA Barcode Reference Library, presented in Supplementary Table 5 (below). Of these, approximately half precede with an a priori taxonomic catalogue, whereas others generate a post-hoc list from the study's results. Most studies take place at taxon, or taxon-by-region scales (country or below), while a few are continental (3) and global (1) in scope. Overall, there is a clear taxon bias toward Insects (19 studies), toward fish among vertebrates (7/10 studies) and a pioneering representation for community assemblages.

		Taxon	A priori catalogue	Catalogue format	Citation
	Insects	Lepidoptera of Germany	Yes	Current regional catalogue	[1]
		Lepidoptera of Australia	No	Survey of a Natural History Collection	[2]
		Butterflies of Malaysia	No	Survey of a Natural History Collection	[3]
		Butterflies of Romania	Yes	Origin not stated	[4]
		Geometrids of Bavaria	Yes	Current regional Catalogue	[5]
		Geometrids of British Columbia	Yes	Composite catalogue, unpublished	[6]
		Noctuidae from Canada	Yes	Composite catalogue, published within citation	[7]
		The Genus Lymantria	Yes	Current catalogue for Genus	[8]
		North American Ephemeroptera	Yes	Current regional catalogue	[9]
		Ephemeroptera, Plecoptera, and Trichoptera of Churchill	Yes	Composite catalogue, published with reference	[10,11]
		Trichoptera of a National Park	Yes	Current Global Catalogue	[12]
		Leaf Beetle community in Costa Rica	No	Regional sampling with post-hoc identification	[13]
		Mosquitoes & Carabid beetles	No	Regional sampling with post-hoc identification	[14]
		Scale Insects	No	Regional sampling, with post-hoc identification	[15]
		Solitary Bees of Ireland	Yes	Unpublished, but recognized catalogue	[16]
		Korean food-pest insects	No	Regional sampling with post-hoc identification	[17]
		Survey of Tephritid Flies	No	Global sampling, with post-hoc identification	[18]
		Chironomidae of Korea	No	Regional sampling with post-hoc identification	[19]
		Syrphidae of a Greek Island	Yes	Catalogue published with reference	[20]
	Spiders	European Spiders	No	Survey of a Natural History Collection	[21]
		Spiders of Churchill	No	Regional sampling with post-hoc identification, catalogue published within citation	[22]
	Vertebrates	Continental-scale Bird Diversity	Yes	Current regional catalogue	[23]
		Neotropical Bats of the Royal Ontario Museum	No	Survey of a Natural History Collection	[24]
		Herpetofauna of Korea	No	Origin not stated	[25]
		Global Fish Diversity	Yes	Current Global Catalogue	[26]
		Marine Fishes of Portugal	No	Regional sampling with post-hoc identification	[27]
		Marine Aquarium Fishes	Yes	Current Global Catalogue	[28]
		Cyprinid Aquarium Fishes	Yes	Current, taxon-related catalogues	[29]
		Marine and Brackish fishes of Argentina	No	Regional sampling with post-hoc identification	[30]
		Freshwater fishes of North America	Yes	Current regional catalogue	[31]
		Marine Fishes of São Paulo, Brazil	Yes	Current regional catalogue	[32]
	Vascular Plants	Plants and Conifers of Wales	Yes	Survey of Natural History Collection	[33]
		Plants of a Panamanian Forest Plot	Yes	Survey of a Natural History Collection	[34]
		Roots of a Panamanian Forest Plot	Yes	Survey of a Natural History Collection	[35]
		Vascular Plants of Churchill	Yes	Current regional catalogue	[36]
	Algae	Red Algae of Canada and France	No	Regional sampling with post-hoc identification	[37]
		Crustose Brown Algae	Unknown		[38]
	Community Assemblages	Freshwater macroinvertebrates	No	Survey of a Natural History Collection	[39]
		Marine macroinvertebrates	Yes	Published catalogue	[40]
		Arctic food web	Yes	Regional sampling with post-hoc identification	[41]
		Island, and reefs of Mo'orea	No	Regional sampling with post-hoc identification	http://www.mooreabiocode.org/

Citations for Supplementary Table 1:

1. Hausmann A, Haszprunar G, Segerer AH, Speidel W, Behounek G, et al. (2011) Now DNA-barcoded: the butterflies and larger moths of Germany. Spixiana 34: 47-58.
2. Hebert PDN, deWaard JR, Zakharov EV, Prosser SWJ, Sones JE, et al. (2013) A DNA 'Barcode Blitz': Rapid Digitization and Sequencing of a Natural History Collection. PLoS ONE 8: e68535.
3. Wilson J-J, Sing K-W, Sofian-Azirun M (2013) Building a DNA Barcode Reference Library for the True Butterflies (Lepidoptera) of Peninsula Malaysia: What about the Subspecies? PLoS ONE 8.
4. Dincă V, Zakharov EV, Hebert PDN, Vila R (2011) Complete DNA barcode reference library for a country's butterfly fauna reveals high performance for temperate Europe. Proceedings of the Royal Society B: Biological Sciences 278: 347-355.
5. Hausmann A, Haszprunar G, Hebert PDN (2011) DNA Barcoding the Geometrid fauna of Bavaria (Lepidoptera): successes, surprises, and questions. PLoS ONE 6.
6. deWaard JR, Hebert PDN, Humble LM (2011) A comprehensive DNA Barcode library for the looper moths (Lepidoptera: Geometridae) of British Columbia, Canada. PLoS ONE 6.
7. Zahiri R, Lafontaine J, Schmidt B, deWaard J, Zakharov E, et al. (2014) Transcontinental Challenge — A Test of DNA Barcode Performance for 1541 Species of Canadian Noctuoidea (Lepidoptera). PLOs ONE, in press.
8. deWaard JR, Mitchell A, Keena MA, Gopurenko D, Boykin LM, et al. (2010) Towards a Global Barcode Library for Lymantria (Lepidoptera: Lymantriinae) Tussock Moths of Biosecurity Concern. PLoS ONE 5.
9. Webb JM, Jacobus LM, Funk DH, Zhou X, Kondratieff B, et al. (2012) A DNA Barcode Library for North American Ephemeroptera: Progress and Prospects. PLoS ONE 7.
10. Zhou X, Adamowicz S, Jacobus L, DeWalt RE, Hebert P (2009) Towards a comprehensive barcode library for arctic life - Ephemeroptera, Plecoptera, and Trichoptera of Churchill, Manitoba, Canada. Frontiers in Zoology 6: 30.
11. Zhou X, Jacobus LM, DeWalt RE, Adamowicz SJ, Hebert PDN (2010) Ephemeroptera, Plecoptera, and Trichoptera fauna of Churchill (Manitoba, Canada): insights into biodiversity patterns from DNA barcoding. Journal of the North American Benthological Society 29: 814-837.
12. Zhou X, Robinson JL, Geraci CJ, Parker CR, Flint OS, et al. (2011) Accelerated construction of a regional DNA-barcode reference library: caddisflies (Trichoptera) in the Great Smoky Mountains National Park. Journal of the North American Benthological Society 30: 131-162.
13. Garcia-Robledo C, Kuprewicz EK, Staines CL, Kress WJ, Erwin TL (2013) Using a comprehensive DNA barcode library to detect novel egg and larval host plant associations in a Cephaloleia rolled-leaf beetle (Coleoptera: Chrysomelidae). Biological Journal of the Linnean Society 110: 189-198.
14. Gibson CM, Kao RH, Blevins KK, Travers PD (2012) Integrative Taxonomy for Continental-Scale Terrestrial Insect Observations. PLoS ONE 7: e37528.
15. Park D-S, Suh S-J, Hebert PDN, Oh H-W, Hong K-J (2011) DNA barcodes for two scale insect families, mealybugs (Hemiptera: Pseudococcidae) and armored scales (Hemiptera: Diaspididae). Bulletin of Entomological Research 101: 429-434.

16. Magnacca KN, Brown MJF (2012) DNA barcoding a regional fauna: Irish solitary bees. Molecular Ecology Resources 12: 990-998.
17. Cho SY, Suh KI, Bae YJ (2013) DNA barcode library and its efficacy for identifying food-associated insect pests in Korea. Entomological Research 43: 253-261.
18. Virgilio M, Jordaens K, Breman FC, Backeljau T, De Meyer M (2012) Identifying Insects with Incomplete DNA Barcode Libraries, African Fruit Flies (Diptera: Tephritidae) as a Test Case. PLoS ONE 7: e31581.
19. Kim S, Song KH, Ree HI, Kim W (2012) A DNA barcode library for Korean Chironomidae (Insecta: Diptera) and indexes for defining barcode gap. Molecules and Cells 33: 9-17.
20. Stahls G, Vujic A, Perez-Banon C, Radenkovic S, Rojo S, et al. (2009) COI barcodes for identification of Merodon hoverflies (Diptera, Syrphidae) of Lesvos Island, Greece. Molecular Ecology Resources 9: 1431-1438.
21. Miller J, Beentjes K, van Helsdingen P, Ijland S (2013) Which specimens from a museum collection will yield DNA barcodes? A time series study of spiders in alcohol. Zookeys 365: 245-261.
22. Blagoev GA, Nikolova NI, Sobel CN, Hebert PDN, Adamowicz SJ (2013) Spiders (Araneae) of Churchill, Manitoba: DNA barcodes and morphology reveal high species diversity and new Canadian records. BMC Ecology 13: 44-44.
23. Lijtmaer DA, Kerr KCR, Barreira AS, Hebert PDN, Tubaro PL (2011) DNA Barcode Libraries Provide Insight into Continental Patterns of Avian Diversification. PLoS ONE 6.
24. Clare EL, Lim BK, Fenton MB, Hebert PDN (2011) Neotropical Bats: Estimating Species Diversity with DNA Barcodes. PLoS ONE 6.
25. Jeong TJ, Jun J, Han S, Kim HT, Oh K, et al. (2013) DNA barcode reference data for the Korean herpetofauna and their applications. Molecular Ecology Resources 13: 1019-1032.
26. Hanner R, Desalle R, Ward RD, Kolokotronis S-O (2011) The Fish Barcode of Life (FISH-BOL) special issue. Mitochondrial DNA 22: 1-2.
27. Costa FO, Landi M, Martins R, Costa MH, Costa ME, et al. (2012) A Ranking System for Reference Libraries of DNA Barcodes: Application to Marine Fish Species from Portugal. PLoS ONE 7: e35858.
28. Steinke D, Zemlak TS, Hebert PDN (2009) Barcoding Nemo: DNA-Based Identifications for the Ornamental Fish Trade. PLoS ONE 4.
29. Collins RA, Armstrong KF, Meier R, Yi YG, Brown SDJ, et al. (2012) Barcoding and Border Biosecurity: Identifying Cyprinid Fishes in the Aquarium Trade. PLoS ONE 7.
30. Mabragana E, de Astarloa JMD, Hanner R, Zhang JB, Castro MG (2011) DNA Barcoding Identifies Argentine Fishes from Marine and Brackish Waters. PLoS ONE 6.
31. April J, Mayden RL, Hanner RH, Bernatchez L (2011) Genetic calibration of species diversity among North America's freshwater fishes. Proceedings of the National Academy of Sciences of the United States of America 108: 10602-10607.
32. Ribeiro AD, Caires RA, Mariguela TC, Pereira LHG, Hanner R, et al. (2012) DNA barcodes identify marine fishes of Sao Paulo State, Brazil. Molecular Ecology Resources 12: 1012-1020.
33. de Vere N, Rich TCG, Ford CR, Trinder SA, Long C, et al. (2012) DNA Barcoding the native flowering plants and conifers of Wales. PLoS ONE 7: e37945.
34. Kress WJ, Erickson DL, Andrew Jones F, Swenson NG, Perez R, et al. (2009) Plant DNA barcodes and a community phylogeny of a tropical forest dynamics plot in Panama.

Proceedings of the National Academy of Sciences of the United States of America 106: 18621-18626.
35. Jones FA, Erickson DL, Bernal MA, Bermingham E, Kress WJ, et al. (2011) The Roots of Diversity: Below Ground Species Richness and Rooting Distributions in a Tropical Forest Revealed by DNA Barcodes and Inverse Modeling. PLoS ONE 6.
36. Kuzmina ML, Johnson KL, Barron HR, Hebert PDN (2012) Identification of the vascular plants of Churchill, Manitoba, using a DNA barcode library. BMC Ecology 12.
37. Gall LLE, Saunders GW (2010) Establishment of a DNA-barcode library for the Nemaliales (Rhodophyta) from Canada and France uncovers overlooked diversity in the species Nemalion helminthoides (Velley) Batters. Cryptogamie Algologie 31: 403-421.
38. Parente MI, Costa FO, Fletcher RL, Saunders GW (2011) A DNA Barcode Library for Crustose Brown Algal Species European Journal of Phycology 46: 66-67.
39. Baird DJ, Pascoe TJ, Zhou X, Hajibabaei M (2011) Building freshwater macroinvertebrate DNA-barcode libraries from reference collection material: formalin preservation vs specimen age. Journal of the North American Benthological Society 30: 125-130.
40. Aylagas E, Borja Á, Rodríguez-Ezpeleta N (2014) Environmental Status Assessment Using DNA Metabarcoding: Towards a Genetics Based Marine Biotic Index (gAMBI). PLoS ONE 9: e90529.
41. Wirta HK, Hebert PDN, Kaartinen R, Prosser SW, Várkonyi G, et al. (2014) Complementary molecular information changes our perception of food web structure. Proceedings of the National Academy of Sciences 111: 1885-1890.
